# Hypothetical Interventions for Falls Among Older Adults: An Application of the Parametric G-Formula

**DOI:** 10.3389/fmed.2021.732136

**Published:** 2021-09-08

**Authors:** Jiaojiao Ren, Guangyou Li, Liju Zhang, Na Zhang, Juan Ren

**Affiliations:** Department of Preventive Medicine, Zhuhai Campus of Zunyi Medical University, Zhuhai, China

**Keywords:** falls, g-formula, hypothetical intervention, older adults, primary prevention

## Abstract

**Introduction:** Falls, which have a higher incidence and mortality due to accidental injuries, are a major global health challenge. The effects of lifestyle factor, health indicator, psychological condition, and functional status interventions on the risk of falls are unknown and the conventional regression model would not adjust for the confounders. This study aimed to evaluate the 4-year risk of falls on the basis of these hypothetical interventions among Chinese older adults.

**Methods:** Data were obtained from 9,692 aged 65 years and over older adults in the China Health and Retirement Longitudinal Study wave, from 2011 to 2015. We used the parametric g-formula to evaluate the risk of falls on the basis of independent hypothetical interventions of sleep duration, social activities, smoking status, drinking status, body mass index (BMI), systolic blood pressure (SBP), vision, depression, activities of daily living (ADL), and their different joint intervention combinations.

**Results:** During the follow-up of 4 years, we documented 1,569 falls. The observed risk of falls was 23.58%. The risk ratios (95% confidence intervals [CIs]) of falls under the intensive hypothetical interventions on increasing sleep duration, participating in more social activities, quit smoking and drinking, reducing BMI and SBP, better vision, alleviating depressive symptoms, and improving ADL capability were 0.93 (0.87–0.96), 0.88 (0.79–0.92), 0.98 (0.95–1.03), 0.97 (0.95–1.02), 0.92 (0.86–1.03), 0.93 (0.87–1.04), 0.86 (0.74–0.91), 0.91 (0.85–0.96), and 0.79 (0.74–0.85), respectively. The feasible and intensive joint hypothetical intervention reduced the 4-year fall risk by 22% (95% CI: 0.52–0.91) and 33% (95% CI: 0.56–0.72), respectively.

**Conclusions:** Hypothetical interventions for increasing sleep duration, participating in more social activities, better vision, alleviating depressive symptoms, and improving ADL capability help protect older adults from falls. Our findings suggest that a combination of lifestyle factors, health indicators, psychological conditions, and functional status may prove to be an effective strategy for preventing falls among older adults.

## Introduction

China is rapidly entering an aging society along with its longer life expectancy. At the end of 2018, 17.9% of the population was aged 60 years and older (1). By 2050, it is estimated that the population aged 60 years and older will increase by 33.6% (2).

Falls, which have a higher incidence and mortality due to accidental injuries, are a major global health challenge. Older adults are more likely to experience falls, which adversely affect their quality of life (3). Falls impose a huge burden on families and healthcare systems in China, including medical and rehabilitation payments, the costs resulting from death, disability, and decline in the ability of daily living (4, 5). Falls increase with advancing age. Previous literature showed that the prevalence of falls among older adults aged over 65 years was 28.0–35.0%, and 32.0–42.0% in those aged over 75 years in China (6). Hence, more effective primary prevention is particularly important for reducing the risk of falls.

Several observational studies have evaluated the risk factors of falls, including biological, psychosocial, socioeconomic, behavioral, and environmental factors (7). The risk of falls in older adults with impaired vision has been reported as 1.7 times higher than that of those with normal vision (8). Less sleep duration or with depressive symptoms are associated with a higher risk of falls in older adults (9, 10). Moreover, body mass index (BMI) and blood pressure are risk factors for falls in older adults (11). However, the existing literatures have the following limitations: most have focused on specific populations (in-hospital or nursing facilities' older adults), and representative community-based older adult study is rare. Moreover, previous studies used either baseline data of fall risk factors or data without adjusting for time-varying confounders (12, 13).

A special methodological challenge is the assessment of an unbiased effect of time-varying exposures in the presence of time-varying confounding factors when confounders are affected by previous exposures (14). In this case, the conventional regression model would not adjust for the confounders and may introduce bias. The parametric g-formula has been developed to deal with such situations and is more effective when exploring the causal effect of intricate interventions (15). In the current study, we performed the parametric g-formula to evaluate the 4-year risk of falls under hypothetical interventions using data from a nationally representative cohort of the China Health and Retirement Longitudinal Study (CHARLS), to provide intervention strategies for older adults aged 65 years and older.

## Materials and Methods

### Study Sample

Data were taken from the CHARLS, which targeted middle-aged and older populations aged 45 years and older, selected through a multistage probability proportional to size sampling. The first baseline survey carried out in 2011 involved 17,705 respondents, covering 150 county-level units and 450 village-level units from 28 provinces (16). It was a longitudinal study, and performed every 2 years for a total of three waves from 2011 to 2015. All the respondents were required to sign informed consent, and the Medical Ethics Committee of Peking University approved the CHARLS study (IRB00001052-11015).

**Figure 1:**
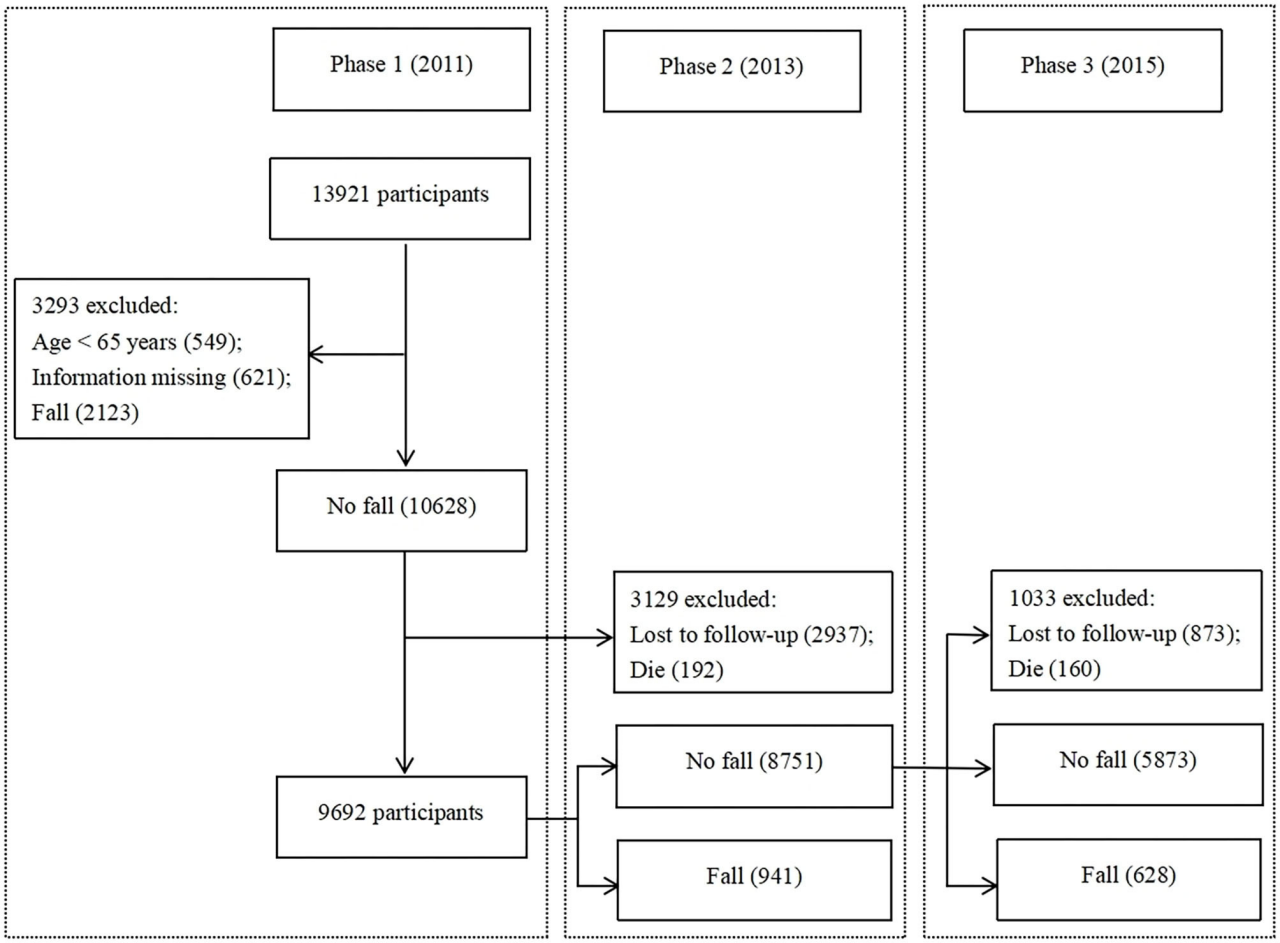
Flow chart of participant enrolment.

This study used the baseline (from the 2011 study) and follow-up (from 2013 and 2015 study) data. We excluded 3,293 participants who at baseline had had falls, incomplete covariate data, or were younger than 65 years (Figure 1). Finally, our study included 9,692 respondents aged 65 years and older.

### Ascertainment of Outcome

In this study, the outcome variable was falls. Participants were asked to provide a “yes” or “no” response to the question “Have you fallen down in the last 2 years?” or “Have you fallen down since the last interview?”

### Ascertainment of Hypothetical Interventions

Our study included four categories of intervention measures: lifestyle factors (sleep duration, social activities, smoking status, and drinking status), health indicators (BMI, systolic blood pressure [SBP], and vision), psychological condition (depression), and functional status (activity of daily living [ADL]). Sleep duration was assessed by the question “During the past month, how many hours of actual sleep did you get at night?.” Social activities were assessed using the following question: “Have you done any of these activities in the last month? (interacted with friends; played Ma-jong, cards, or went to a community club; provided help to family, friends, or neighbors; took part in a community-related organization; went to a sport, social, or other kind of club; done voluntary or charity work; cared for a sick or disabled adult; attended an educational or training course; stock investment; used the Internet; and others). According to the responses, participants who participated in one or more items were defined as “participating in social activities,” otherwise they were regarded as “not participating in social activities.” Smoking status was assessed by the question “Have you ever chewed tobacco, smoked a pipe, smoked self-rolled tobacco, or smoked cigarettes/cigars?” (yes, no, or quit). Drinking status was assessed by the question “Did you drink any alcoholic beverages in the past year. How often?” (drink alcohol more than once a month, drink alcohol but less than once a month, and do not drink alcohol). BMI was calculated and categorized as: <18.5, 18.5–23.9, and ≥24.0 kg/m^2^. SBP was calculated as the average of three blood pressure measurements using a sphygmomanometer. The vision level of the participants was assessed by the following question: “Would you say your eyesight for seeing things up close is excellent, very good, good, fair, or poor?.” We categorized “excellent, very good, and good” as “good,” others as “fair or poor.” Depressive symptoms were measured by the Center for Epidemiological Studies Depression Scale (CES-D-10), which consisted of 10 items with 4 response options (rarely or none of the times, some or a few times, occasionally or a moderate amount of time, and most or all of the time). The values for the 4 options ranged from 0 to 3. A cutoff score of ≥10 was defined as having depressive symptoms in participants (17). ADL was measured using the ADL scale, including dressing, bathing, eating, getting out of bed, using the toilet, controlling urination and defecation, doing chores, preparing hot meals, shopping, managing money, and taking medications (18). The responses comprised of four options (having no difficulty, having difficulty but can still do it, having difficulty and need help, and cannot do it). In the participants, we regarded having the ability to complete all these items without difficulty as being ADL independent, and having difficulty with any item as having restricted ADL.

### Ascertainment of Covariates

The sociodemographic characteristics included age (60–74 years or ≥75 years), sex (men or women), education level (no more than elementary school, middle school, or high school and above), and marital status (married, widowed, or others). The time-varying variables included residence (urban or rural), chronic condition (lived with one or more of the 14 chronic conditions diagnosed by a doctor in the previous 6 months), self-assessed health status (good, fair, or poor), and cognitive function. Cognitive function was measured using three tests: episodic memory, figure drawing, and Telephone Interview for Cognitive Status (TICS). The episodic memory, figure drawing, and TICS scores ranged from 0 to 10, 0 to 1, and 0 to 10, respectively. The cognitive function score was the sum of the three test scores and ranged from 0 to 21 (19).

### Hypothetical Interventions on Risk Factors for Falls

We presented nine feasible and nine intensive hypothetical interventions, and the joint interventions. The feasible interventions were determined in view of evidence from randomized controlled trials (RCTs) or clinical guidelines (20–22). These were: 50% of individuals had increased sleep duration from <8 to 8 h; all individuals participated in social activities every week; 13% of smokers quit smoking; 20% of drinkers quit drinking; all individuals maintained BMI ≤ 27.9 kg/m^2^; all individuals maintained SBP <140 mmHg; 50% of individuals had fair or good vision; 50% of depressed individuals were no longer depressive; and 50% of individuals did not have restricted ADL. The intensive interventions were as follows: all individuals had increased sleep duration from <8 to 8 h; all individuals participated in social activities every day; all smokers quit smoking; all drinkers quit alcohol; all individuals maintained BMI ≤ 23.9 kg/m^2^; all individuals maintained SBP <120 mmHg; all individuals had fair or good vision; all individuals were not depressed; and all individuals did not have restricted ADL.

### Statistical Analysis

The parametric g-formula, which indicated the standardization generalization for time-varying exposures and confounders, was performed to estimate the 4-year cumulative risk of falls on the basis of hypothetical interventions (23). We conducted regression models for the time-varying covariates (deaths and falls). Furthermore, we simulated the risk of falls under each intervention through these models by the following steps: using the observed values of covariates at baseline; using a parametric model to evaluate the combined distribution of time-varying covariates; intervening by setting the value of the covariate to the values based on the hypothetical intervention; and assessing the predicted risk of falls and death by the new values.

The estimated fall risks were compared under different interventions to the 4-year risk under no intervention to calculate the risk ratios (RRs) and risk differences (RDs) for the population. We conducted subgroup analyses to assess potential effects by sex (men or women), and age (60–74 years or ≥75 years). We also conducted sensitivity analyses, in which we changed the order of the time-varying covariates under interventions in the model and excluded the participants who reported falls with SBP <100 mmHg. We used nonparametric bootstrapping with 500 samples to estimate the 95% confidence intervals (CIs). The proportion of individuals who were intervened at any period, as well as the average proportion of individuals intervened at each 2-yearly period, were calculated. All analyses were performed using SAS (version 9.4; SAS Institute, Cary, NC, USA).

## Results

During the follow-up of 4 years, we documented 1,569 falls. Of the 9,692 eligible participants, the mean age was 73.2 (8.6) years, and 4,900 (50.6%) were women. Overall, 5,373 individuals (55.4%) participated in social activities, and over half (53.1%) had chronic disease. Only 15.2% had restricted ADL, and ~30.0% had good vision. The mean scores of depression and cognitive function were 10.2 and 11.0, respectively. The mean sleep duration was 6.2 h. The characteristics of the eligible participants are shown in Table 1.

**Table 1:** Demographic characteristics of study participants (2011).

**Characteristics**	***N***	**%**
**Age, years**		
65–74	7,771	80.2
≥75	1,921	19.8
**Sex**, ***n*****(%)**		
Men	4,792	49.4
Women	4,900	50.6
**Level of education**, ***n*****(%)**		
No more than elementary school	3,835	39.6
Middle school	3,751	38.7
High school and above	2,106	21.7
**Marital status**, ***n*****(%)**		
Married	8,439	87.1
Widowed	1,035	10.7
Others	218	2.2
**Residence**, ***n*****(%)**		
Urban	6,022	62.1
Rural	3,670	37.9
**Smoking status**, ***n*****(%)**		
Yes	849	8.8
No	8,843	91.2
**Drinking status**, ***n*****(%)**		
Yes	3,576	36.9
No	6,116	63.1
**Participating in social activities**, ***n*****(%)**		
Yes	5,373	55.4
No	4,319	44.6
**Chronic condition**, ***n*****(%)**		
Yes	5,147	53.1
No	4,545	46.9
**Self-assessed health status**, ***n*****(%)**		
Good	1,566	16.2
Fair	5,869	60.6
Poor	2,257	23.2
**Restricted ADL**, ***n*****(%)**		
Yes	1,475	15.2
No	8,217	84.8
**Vision**, ***n*****(%)**		
Good	2,850	29.4
Fair	4,485	46.3
Poor	2,357	24.3
**BMI**, ***n*****(%)**		
<18.5 kg/m^2^	2,370	24.4
18.5–23.9 kg/m^2^	4,884	50.4
≥24.0 kg/m^2^	2,438	25.2
**SBP**, ***n*****(%)**		
<120 mmHg	3,343	34.5
121–139 mmHg	3,620	37.4
>140 mmHg	2,729	28.2
Depression score, mean (SD)	10.2	4.5
Sleep duration, mean (SD), h	6.2	2.1
Cognitive function score, mean (SD)	11.0	3.4

*ADL, Activity of daily living; BMI, Body Mass Index; SBP, Systolic Blood Pressure*.

The simulated 4-year risk of falls under no intervention was 22.21% (95% CI: 20.41–24.20), similar to the observed risk of 23.58%. The model performed well-based on the relatively small error between the simulated and observed values of each covariate in this study (Table 2).

**Table 2:** The comparison of actual observation and simulated average values.

	**2011**			**2013**			**2015**		
	**Observed data**	**Simulated data**	**Relative error (%)**	**Observed data**	**Simulated data**	**Relative error (%)**	**Observed data**	**Simulated data**	**Relative error (%)**
Residence	0.34	0.34	1.00	0.34	0.34	1.00	0.32	0.34	6.90
Chronic condition	1.31	1.32	1.90	1.56	1.71	9.10	1.14	1.46	28.10
Self-assessed health status	1.95	1.95	0.30	1.96	1.95	0.40	1.96	1.80	7.80
Smoking	0.72	0.72	0.40	0.72	0.73	1.50	0.70	0.73	6.50
Drinking	0.60	0.60	0.40	0.59	0.60	1.50	0.61	0.63	4.50
Social activities	1.14	1.13	1.40	1.19	1.15	3.40	1.17	1.04	11.60
Vision	0.76	0.76	0.40	0.75	0.74	1.50	0.78	0.76	4.60
Body mass index	1.56	1.56	0.40	1.62	1.62	0.50	1.62	1.61	0.50
Systolic blood pressure	2.02	2.01	0.40	2.06	2.05	0.90	2.00	2.07	3.50
Activity of daily living	0.12	0.12	0.40	0.17	0.18	16.70	0.20	0.26	34.50
Depression	9.70	9.74	0.50	7.96	8.82	10.50	8.92	10.26	16.00
Sleep duration	6.33	6.34	0.10	6.20	6.13	1.60	6.43	6.20	3.70
Cognitive function	0.40	0.40	0.20	0.62	0.68	8.00	0.43	0.52	20.50

The feasible interventions can substantially reduce the risk of falls. Specifically, compared to no intervention, increasing sleep duration to 8 h; participating in social activities every week; improving the vision, depression condition, and ADL capability in 50% of individuals would reduce fall risk by 4.0, 5.0, 4.0, 4.0, and 15%, respectively; whereas 13% of smokers quit smoking, 20% of drinkers quit drinking, and reducing BMI and SBP did not substantially alter the fall risk (Table 3).

**Table 3:** Risk of fall under feasible hypothetical interventions (CHARLS 2011–2015).

**No.**	**Intervention**	**4-year risk of fall (%) (95% CI)^a^**	**RR (95% CI)^b^**	**RD (95% CI)^b^**	**Cumulative intervention (%)**	**Average intervention (%)**
0	No intervention	22.21 (20.41, 24.20)	1.00	0.00	0	0
1	Sleep duration increased to 8 h for 50% of individuals	21.35 (20.44, 23.58)	0.96 (0.92, 0.98)	−0.86 (−0.95, −0.43)	76	36
2	Participating in social activities every week	21.20 (17.34, 24.99)	0.95 (0.70, 0.99)	−1.01 (−1.12, −0.49)	98	90
3	13% of smokers quit smoking	21.86 (21.18, 24.01)	0.98 (0.96, 1.02)	−0.35 (−0.51, 0.85)	21	7
4	20% of drinkers quit drinking	21.77 (21.04, 23.85)	0.97 (0.96, 1.02)	−0.44 (−0.62, 1.02)	36	22
5	BMI reduced to ≤ 27.9 kg/m^2^	22.10 (21.54, 23.68)	0.99 (0.98, 1.03)	−0.11 (−0.26, 0.52)	30	11
6	SBP reduced to <140 mmHg	21.96 (21.23, 24.12)	0.98 (0.97, 1.03)	−0.25 (−0.43, 0.73)	45	20
7	50% of individuals intervened as fair or good vision	21.46 (20.62, 23.17)	0.96 (0.92, 0.99)	−0.75 (−0.87, −0.40)	80	48
8	50% of depressed individuals were not depressive	21.40 (20.84, 23.97)	0.96 (0.95, 0.98)	−0.81 (−0.94, −0.42)	37	18
9	ADL for 50% of individuals was not restricted	20.21 (19.03, 21.46)	0.85 (0.82, 0.89)	−2.00 (−2.52, −1.03)	36	21

a *The observed risk is 23.58%*.

b *Estimated using the parametric g-formula with covariates: age, sex, marital status, education level, and marital status; time-varying covariates: residence, chronic condition, self-assessed health status, cognitive function, sleep duration, social activities, smoking status, drinking status, BMI, SBP, vision, depression, and ADL*.

*ADL, Activity of daily living; BMI, Body Mass Index; SBP, Systolic Blood Pressure; RR, Risk Ratio; RD, Risk Difference; CI, Confidence Interval*.

Under intensive interventions for the same interventions, increasing sleep duration to 8 h and the frequency of participation in social activities to every day, and improving the vision, depression condition, and ADL capability in 100% of individuals reduced the 4-year fall risk by 7.0, 12.0, 14.0, 9.0, and 21%, respectively. Other intensive interventions did not substantially change the risk of falls (Table 4). Subgroup analyses showed the estimated effect of intensive intervention stratified by sex and age. Compared to no intervention, women had a higher risk of falling than men. In addition to the intervention for sleep duration, other interventions were more effective in women than in men. However, the heterogeneity test showed that the effects of all the intensive interventions in men and women were not statistically significant (Table 5). Furthermore, participants aged 65–74 years had a lower risk of falls than those aged ≥75 years under no intervention. For the intervention of sleep duration and the frequency of social activities, fall risk in the 65–74 years group would be better intervened in than that in the ≥75 years group. Similarly, there were no differences between being aged 65–74 years and ≥75 years under intensive interventions (Table 6).

**Table 4:** Risk of fall under intensive hypothetical interventions (CHARLS 2011–2015).

**No.**	**Intervention**	**4-year risk of fall (%) (95% CI)^a^**	**RR (95% CI)^b^**	**RD (95% CI)^b^**	**Cumulative intervention (%)**	**Average intervention (%)**
0	No intervention	22.21 (20.41, 24.20)	1.00	0.00	0	0
1	Sleep duration increased to 8 h for all individuals	21.12 (20.44, 23.58)	0.93 (0.87, 0.96)	−1.09 (−1.21, −0.12)	95	71
2	Participating in social activities every day	20.13 (19.31, 22.67)	0.88 (0.79, 0.92)	−2.08 (−4.34, −1.15)	98	84
3	All smokers quit smoking	21.57 (20.89, 23.46)	0.98 (0.95, 1.03)	−0.64 (−0.76, 0.19)	48	18
4	All drinkers quit drinking	21.30 (19.97, 23.24)	0.97 (0.95, 1.02)	−0.91 (−1.03, 0.24)	49	20
5	BMI reduced to ≤ 23.9 kg/m^2^	21.01 (20.34, 22.92)	0.92 (0.86, 1.03)	−1.20 (−1.31, 0.45)	42	17
6	SBP reduced to <120 mmHg	21.04 (20.22, 22.84)	0.93 (0.87, 1.04)	−1.17 (−1.30, 0.68)	78	45
7	All individuals intervened as fair or good vision	20.42 (20.03, 23.52)	0.86 (0.74, 0.91)	−1.79 (−1.95, −0.76)	83	49
8	All depressed individuals were not depressive	20.97 (18.84, 23.31)	0.91 (0.85, 0.96)	−1.24 (−1.37, −0.23)	71	36
9	ADL for all individuals was not restricted	18.11 (17.06, 19.42)	0.79 (0.74, 0.85)	−4.10 (−5.72, −3.15)	86	51

a *The observed risk is 23.58%*.

b *Estimated using the parametric g-formula with covariates: age, sex, marital status, education level, and marital status; time-varying covariates: residence, chronic condition, self-assessed health status, cognitive function, sleep duration, social activities, smoking status, drinking status, BMI, SBP, vision, depression, and ADL*.

*ADL, Activity of daily living; BMI, Body Mass Index; SBP, Systolic Blood Pressure; RR, Risk Ratio; RD, Risk Difference; CI, Confidence Interval*.

**Table 5:** Risks of falls under intensive interventions by sex.

**No.**	**Intervention**	**Men**		**Women**		**Heterogeneity**
		**4-year risk of fall (%) (95% CI)^a^**	**RR (95% CI)^b^**	**4-year risk of fall (%) (95% CI)^c^**	**RR (95% CI)^b^**	***P*** **-value**
0	No intervention	20.64 (19.22, 22.31)	1.00	25.27 (24.21, 26.73)	1.00	–
1	Sleep duration increased to 8 h for all individuals	18.68 (17.21, 20.16)	0.91 (0.88, 0.97)	24.15 (22.96, 26.01)	0.96 (0.91, 1.00)	0.580
2	Participating in social activities every day	19.67 (16.41, 23.34)	0.97 (0.72, 1.12)	21.89 (22.96, 26.01)	0.85 (0.70, 1.05)	0.644
3	All smokers quit smoking	20.21 (19.43, 22.89)	0.99 (0.98, 1.03)	24.20 (22.59, 25.89)	0.96 (0.93, 1.07)	0.606
4	All drinkers quit alcohol	20.12 (19.32, 23.12)	0.98 (0.97, 1.03)	23.89 (22.04, 25.71)	0.95 (0.92, 1.05)	0.581
5	BMI reduced to ≤ 23.9 kg/m^2^	20.09 (19.30, 23.08)	0.98 (0.96, 1.02)	23.54 (21.96, 25.45)	0.95 (0.92, 1.04)	0.523
6	SBP reduced to <120 mmHg	20.01 (19.17, 23.02)	0.98 (0.95, 1.02)	23.26 (21.43, 25.22)	0.94 (0.91, 1.03)	0.519
7	All individuals intervened as fair or good vision	19.75 (18.52, 20.48)	0.97 (0.93, 0.99)	24.38 (23.03, 26.08)	0.97 (0.94, 0.98)	0.678
8	All depressed individuals were not depressive	19.36 (17.92, 20.47)	0.95 (0.91, 0.96)	23.51 (22.06, 24.87)	0.92 (0.90, 0.96)	0.458
9	ADL for all individuals was not restricted	16.84 (15.67, 19.03)	0.81 (0.76, 0.89)	18.12 (15.54, 20.73)	0.70 (0.61, 0.82)	0.302

a *The observed risk is 21.60%*.

b *Estimated using the parametric g-formula with covariates: age, marital status, education level, and marital status; time-varying covariates: residence, chronic condition, self-assessed health status, cognitive function, sleep duration, social activities, smoking status, drinking status, BMI, SBP, vision, depression, and ADL*.

c *The observed risk is 26.23%*.

*ADL, Activity of daily living; BMI, Body Mass Index; SBP, Systolic Blood Pressure; RR, Risk Ratio; CI, Confidence Interval*.

**Table 6:** Risks of falls under single intensive interventions by age.

**No.**	**Intervention**	**60–74 years**		**≥75 years**		**Heterogeneity**
		**4-year risk of fall (%) (95% CI)^a^**	**RR (95% CI)^b^**	**4-year risk of fall (%) (95% CI)^c^**	**RR (95% CI)^b^**	***P*** **-value**
0	None	22.38 (21.42, 24.49)	1.00	24.19 (22.90, 26.08)	1.00	–
1	Sleep duration increased to 8 h for all individuals	20.54 (19.02, 22.94)	0.91 (0.87, 1.08)	23.25 (21.11, 24.52)	0.96 (0.90, 0.98)	0.481
2	Participating in social activities every day	16.47 (15.02, 22.75)	0.73 (0.62, 1.02)	25.10 (19.17, 31.88)	1.02 (0.78, 1.35)	0.420
3	All smokers quit smoking	21.94 (20.17, 24.10)	0.97 (0.93, 1.04)	23.98 (22.70, 25.22)	0.97 (0.94, 1.08)	0.543
4	All drinkers quit alcohol	21.73 (20.03, 24.01)	0.95 (0.91, 1.03)	23.74 (22.46, 25.01)	0.98 (0.93, 1.07)	0.502
5	BMI reduced to ≤ 23.9 kg/m^2^	21.67 (19.89, 23.84)	0.95 (0.91, 1.03)	23.45 (22.13, 24.89)	0.97 (0.92, 1.07)	0.535
6	SBP reduced to <120 mmHg	21.45 (19.72, 23.56)	0.94 (0.90, 1.02)	23.36 (22.07, 24.68)	0.97 (0.92, 1.06)	0.591
7	All individuals intervened as fair or good vision	21.37 (20.29, 24.23)	0.94 (0.91, 1.04)	23.23 (21.90, 24.53)	0.96 (0.91, 0.98)	0.668
8	All depressed individuals were not depressive	21.15 (20.15, 24.05)	0.93 (0.91, 0.97)	22.57 (21.36, 24.86)	0.94 (0.89, 0.97)	0.707
9	ADL for all individuals was not restricted	16.20 (15.27, 17.70)	0.70 (0.66, 0.77)	19.36 (16.35, 21.18)	0.79 (0.70, 0.83)	0.125

a *The observed risk is 22.04%*.

b *Estimated using the parametric g-formula with covariates: sex, marital status, education level, and marital status; time-varying covariates: residence, chronic condition, self-assessed health status, cognitive function, sleep duration, social activities, smoking status, drinking status, BMI, SBP, vision, depression, and ADL*.

c *The observed risk is 25.93%*.

*ADL, Activity of daily living; BMI, Body Mass Index; SBP, Systolic Blood Pressure; RR, Risk Ratio; CI, Confidence Interval*.

The results of the analyses for the joint interventions are presented in Table 7, which excluded four interventions (smoking status, drinking status, BMI, and SBP) that were not statistically significant in the single intervention analysis. All intensive combination interventions could significantly reduce the risk of falls in older adults. The five complicated intensive interventions, which included increasing sleep duration and the frequency of participating in social activities; and improving the vision, depression condition, and ADL capability reduced the risk of falls the most (RR: 0.67, 95% CI: 0.56–0.72). These were followed by interventions that combined increasing sleep duration and the frequency of participating in social activities, and improving the vision and depression condition (RR: 0.74, 95% CI: 0.62–0.79); increasing sleep duration combined with the frequency of participating in social activities and improving the vision (RR: 0.78, 95% CI: 0.65–0.86); and increasing sleep duration combined with the frequency of participating in social activities (RR: 0.81, 95% CI: 0.71–0.90). The sensitivity analysis, which examined whether the order of the variables and the participants who reported falls with SBP <100 mmHg could affect the results, revealed that the RR and RD did not change materially (Tables 8, 9).

**Table 7:** Effect of joint hypothetical interventions on 4-years fall risk (CHARLS 2011–2015).

**No.**	**Intervention**	**4-year risk of fall (%) (95% CI)^a^**	**RR (95% CI)^b^**	**RD (95% CI)^b^**	**Cumulative intervention (%)**	**Average intervention (%)**
0	No intervention	22.21 (20.41, 24.20)	1.00	0.00	0	0
**Feasible joint interventions**						
1	Sleep duration, and social activities	20.96 (15.01, 24.25)	0.92 (0.74, 1.04)	−1.25 (−6.76, 1.05)	100	94
2	Sleep duration, social activities, and vision	20.57 (14.86, 23.74)	0.89 (0.65, 1.04)	−1.64 (−7.19, 1.12)	100	95
3	Sleep duration, social activities, vision, and depression	20.24 (14.34, 23.28)	0.86 (0.61, 1.03)	−1.97 (−7.54, 0.96)	100	95
4	Sleep duration, social activities, vision, depression, and ADL	19.87 (13.59, 22.57)	0.78 (0.52, 0.91)	−2.34 (−7.91, −1.80)	100	96
**Intensive joint interventions**						
1	Sleep duration, and social activities	19.14 (17.23, 21.25)	0.81 (0.71, 0.90)	−3.07 (−5.23, −0.74)	100	97
2	Sleep duration, social activities, and vision	18.56 (16.63, 20.81)	0.78 (0.65, 0.86)	−3.65 (−5.92, −1.38)	100	97
3	Sleep duration, social activities, vision, and depression	17.53 (15.56, 19.72)	0.74 (0.62, 0.79)	−4.68 (−7.04, −2.45)	100	98
4	Sleep duration, social activities, vision, depression, and ADL	16.42 (14.40, 18.61)	0.67 (0.56, 0.72)	−5.79 (−8.21, −3.76)	100	98

a *The observed risk is 23.58%*.

b *Estimated using the parametric g-formula with covariates: age, sex, marital status, education level, and marital status; time-varying covariates: residence, chronic condition, self-assessed health status, cognitive function, sleep duration, social activities, smoking status, drinking status, BMI, SBP, vision, depression, and ADL*.

*ADL, Activity of daily living; RD, risk difference; CI, Confidence Interval*.

**Table 8:** Sensitivity analyses for risks of falls by changing the order of time-varying covariates under interventions.

**Interventions**	**4-year risk of fall (%) (95% CI)**	**RR (95% CI)^a^**
None	22.03 (20.41, 24.20)	1.00
**Feasible intervention**		
Sleep duration increased to 8 h for 50% of individuals	21.15 (20.24, 23.28)	0.94 (0.91, 0.97)
Participating in social activities every week	21.32 (17.54, 25.02)	0.96 (0.72, 0.99)
13% of smokers quit smoking	21.70 (20.97, 23.81)	0.98 (0.96, 1.02)
20% of drinkers quit drinking	21.78 (21.05, 23.89)	0.98 (0.96, 1.03)
BMI reduced to ≤ 27.9 kg/m^2^	22.06 (21.46, 23.58)	0.99 (0.98, 1.04)
SBP reduced to <140 mmHg	21.87 (21.13, 23.25)	0.98 (0.97, 1.03)
50% of individuals intervened as fair or good vision	21.23 (20.42, 22.87)	0.96 (0.92, 0.99)
50% of depressed individuals were not depressive	21.44 (20.74, 23.40)	0.97 (0.96, 0.98)
ADL for 50% of individuals was not restricted	20.29 (19.13, 21.84)	0.86 (0.83, 0.89)
**Intensive intervention**		
Sleep duration increased to 8 h for all individuals	20.97 (20.13, 23.28)	0.91 (0.85, 0.95)
Participating in social activities every day	20.15 (19.34, 22.69)	0.88 (0.78, 0.91)
All smokers quit smoking	21.69 (21.01, 23.57)	0.97 (0.93, 1.02)
All drinkers quit drinking	21.20 (19.77, 23.04)	0.96 (0.94, 1.05)
BMI reduced to ≤ 23.9 kg/m^2^	21.03 (20.36, 22.95)	0.92 (0.86, 1.03)
SBP reduced to <120 mmHg	21.01 (20.18, 22.81)	0.92 (0.87, 1.04)
All individuals intervened as fair or good vision	20.40 (20.07, 23.47)	0.86 (0.73, 0.90)
All depressed individuals were not depressive	20.95 (18.82, 23.29)	0.91 (0.84, 0.96)
ADL for all individuals was not restricted	18.31 (17.45, 19.71)	0.81 (0.72, 0.83)

a *The order of time-varying covariates was residence, cognitive function, smoking status, drinking status, social activities, self-assessed health status, vision, chronic condition, ADL, BMI, depression, SBP, and sleep duration; the order of time-varying covariates in this study was residence, chronic condition, self-assessed health status, cognitive function, sleep duration, social activities, smoking status, drinking status, BMI, SBP, vision, depression, and ADL*.

*ADL, Activity of Daily Living; BMI, Body Mass Index; SBP, Systolic Blood Pressure; RR, Risk Ratio; CI, Confidence Interval*.

**Table 9:** Sensitivity analyses for risks of falls by excluding the participants who reported falls with SBP < 100 mmHg under interventions.

**Interventions^a^**	**4-year risk of fall (%) (95% CI)**	**RR (95% CI)**
None	21.86 (20.12, 23.94)	1.00
**Feasible intervention**		
Sleep duration increased to 8 h for 50% of individuals	21.01 (20.04, 22.96)	0.93 (0.89, 0.96)
Participating in social activities every week	21.18 (17.21, 24.80)	0.95 (0.75, 0.98)
13% of smokers quit smoking	21.56 (20.66, 23.70)	0.97 (0.94, 1.01)
20% of drinkers quit drinking	21.60 (20.89, 23.62)	0.97 (0.94, 1.02)
BMI reduced to ≤ 27.9 kg/m^2^	21.91 (21.26, 23.36)	0.98 (0.96, 1.03)
SBP reduced to <140 mmHg	21.72 (20.91, 23.05)	0.97 (0.96, 1.03)
50% of individuals intervened as fair or good vision	21.03 (20.26, 22.62)	0.95 (0.90, 0.98)
50% of depressed individuals were not depressive	21.32 (20.65, 23.27)	0.95 (0.94, 0.98)
ADL for 50% of individuals was not restricted	20.14 (18.97, 21.78)	0.84 (0.81, 0.87)
**Intensive intervention**		
Sleep duration increased to 8 h for all individuals	20.81 (20.01, 23.12)	0.90 (0.83, 0.95)
Participating in social activities every day	20.01 (19.14, 22.49)	0.87 (0.76, 0.90)
All smokers quit smoking	21.54 (20.87, 23.36)	0.96 (0.91, 1.01)
All drinkers quit drinking	21.04 (19.55, 22.84)	0.95 (0.93, 1.04)
BMI reduced to ≤ 23.9 kg/m^2^	20.93 (20.16, 22.80)	0.92 (0.85, 1.02)
SBP reduced to <120 mmHg	20.85 (20.02, 22.73)	0.91 (0.87, 1.03)
All individuals intervened as fair or good vision	20.24 (19.92, 23.32)	0.85 (0.71, 0.88)
All depressed individuals were not depressive	20.81 (18.68, 23.13)	0.90 (0.82, 0.96)
ADL for all individuals was not restricted	18.17 (17.25, 19.52)	0.81 (0.70, 0.82)

a *ADL, Activity of daily living; BMI, body mass index; SBP, Systolic blood pressure; RR, risk ratio; CI, confidence interval*.

## Discussion

Our study found that a feasible hypothetical intervention of a functional status factor (ADL) was associated with decreased fall risk (15%) during the 4-year follow-up in Chinese older adults, whereas intensive hypothetical intervention decreased fall risk (21%). A combination of feasible modifications (sleep duration, social activities, vision, depression, and ADL) led to a 22% reduction in the risk of falls, and a more intensive combined intervention could reduce the risk of falls by 33%. Recently, a meta-analysis of RCTs on preventing falls in community-dwelling older adults also showed that multifactorial interventions could reduce the risk of falls by 34 and 28% in the high-risk and healthy groups, respectively (24). This revealed that the reduced fall rates in this study was very close to that of our study.

The results of our hypothetical interventions for improving the effect of ADL capability on falls, which would reduce the 4-year fall risk, are in line with those of previous studies. Prospective studies reported that older adults with higher ADL capability had a lower risk of falls, especially for those with a higher frequency of shopping activities and doing chores (25, 26). It has been suggested that restricted ADL is associated with decreased strength, balance, and endurance (27, 28). As a result, the risk of falls increases. The present study also found that the association between being aged ≥75 years and fall risk became stronger. This may be because the decline in ADL capability increases with age (29).

We found that better vision could protect older adults from falls. Considerable studies have shown that older adults with impaired vision have a higher risk of falls (30, 31). Among explanations regarding the reasons why impaired vision may be associated with increased risk of falls, most are related to processes that are implicated in body balance, such as the coordination of visual and vestibular system, muscle strength, and reaction time.

The hypothetical interventions of participating in social activities from our results could reduce the 4-year risk of falls in older adults. A prospective study provided clear evidence of the association between participating in more social activities and a lower risk of falls among older adults (32). In a meta-analysis of RCTs, dance-based mind-motor activities were significantly associated with a 37% reduced risk of falls among older adults (33). Dance offers older people an opportunity for greater social activities, thereby contributing to falls prevention.

The significant risk reduction for falls by alleviating depressive symptoms in this study is consistent with previous evidence connecting depressive symptoms with falls (34, 35). A prospective study found that alleviating depressive symptoms was associated with a decline in fall risk in a large sample of community-dwelling older people (36).

The hypothetical intervention of extending the sleep duration to 8 h can effectively reduce the risk of falls among older adults. Existing literature reveals that participants who have a sleep duration of ≤ 5 h are more likely to report falls, whereas no association was found between sleep duration > 8 h and falls (37). However, a meta-analysis of observational studies concluded that an approximately U-shaped curve was observed between sleep duration and falls; that is, both short and long sleep durations are significantly associated with falls (38). Since only 4.21% of the included samples had sleep durations longer than 8 h, an intervention analysis on the impact of excessive sleep duration on falls was not conducted in this study. Future research need to be launched on an intervention with longer sleep duration to further explore the effect of sleep duration on falls.

We did not found a significant effect of cessation of smoking or alcohol on falls. There was a hysteresis effect of the hypothetical intervention of smoking and drinking, which resulted in health benefits that could not be observed in the short term or during the follow-up period. Similarly, a study of stroke showed that the excess risk of stroke among former smokers disappeared from 2 to 4 years after cessation (39). In addition, an effect of reducing BMI and SBP on falls was not found. A longitudinal population-based survey suggested that obesity (BMI ≥ 28.0 kg/m^2^) appeared to be associated with a greater risk of falls in older adults (40). Possible mechanisms include lower levels of physical activity, postural balance, and vitamin D deficiency (41). In our study, reducing the BMI to overweight or normal levels did not significantly decrease the risk of falls. One plausible explanation is that the proportion of overweight or obese participants was low, and the hypothetical intervention result was not obvious. The relationship between SBP and falls is complex. Evidence of SBP and the risk of falls in older adults are inconsistent. While an observational study has demonstrated that SBP is a significant risk factor for falls (42), other studies showed no association (43, 44). The recent studies concluded that orthostatic hypotension and low SBP were significantly positively correlated with falls in older adults (45, 46). However, the sensitivity analysis in our study showed no substantial change when we excluded the participants who reported falls with SBP < 100 mmHg during the follow-up.

Our study has several strengths, including its population-based longitudinal design, standardized survey methods, and longer follow-up time. We applied the parametric g-formula with adjustment for time-varying confounders by risk factors of falls and simulated interventions on lifestyle factors, health indicators, psychological conditions, and functional status. However, there were several limitations to this study. First, the assumptions for the observational study were no model misspecification, no measurement error, and no unmeasured confounding, which determined the validity of the parametric g-formula in our study. We have adjusted for multiple risk factors to alleviate the problems of no unmeasured confounding factors that are inevitable in an observational research. Second, the simulated data from the parametric g-formula was similar to the observed data, which revealed the necessary condition of the absence of model misspecification, and the results of the sensitivity analyses were robust across different specifications. Third, our study assumed that the counterfactual result of each scenario should be the same with the results observed under the observed exposure history, which requires consistency. This assumption may have been met for sleep duration and social activities, but less likely for BMI, SBP, depression, and ADL. Hence, the hypothetical effects of BMI, SBP, depression, and ADL should be interpreted as the influence of the combination of factors that can change these interventions. Fourth, we could not provide information on subdivision of falls (recurrent, unexplained, and injurious falls), polypharmacy, and orthostatic hypotension because of the lack of these information in the survey. Finally, in the parametric g-formula in this study, the risk was standardized based on the distribution of confounding factors, and caution should be exercised when generalizing these results to other populations.

## Conclusion

This study found that hypothetical interventions for increasing sleep duration, participating in more social activities, better vision, alleviating depressive symptoms, as well as improving ADL capability, were beneficial to protect older adults from falls by applying a parametric g-formula on the CHARLS data. Our findings suggest that a combination of lifestyle factors, health indicators, psychological conditions, and functional status may prove to be an effective strategy for preventing falls among older adults.

## Data Availability Statement

The raw data supporting the conclusions of this article will be made available by the authors, without undue reservation.

## Ethics Statement

The studies involving human participants were reviewed and approved by Medical Ethics Committee of Peking University (IRB00001052-11015). The patients/participants provided their written informed consent to participate in this study.

## Author Contributions

JiR and GL contributed to conception, design, data acquisition and interpretation, and critically revised the manuscript. LZ contributed to conception, data interpretation, and performed all statistical analyses. NZ contributed to conception and drafted manuscript. JuR contributed to data cleaning and critically revised the manuscript. All authors gave their final approval and agree to be responsible for all aspects of the work.

## Funding

This work was supported by the Science and Technology Program of Guizhou Province (grant number ZK[2021]495), the Doctoral Research Initiation Fund Project of Zunyi Medical University (grant number F-947), the Project of philosophy and social science planning in 2019–2020 in Zhuhai (grant number 2019ZC106), and the Sichuan Provincial Education Department humanities and social sciences key research base-Sichuan Hospital Management and Development Research Center of Southwest Medical University (grant number SCYG2019-31).

## Conflict of Interest

The authors declare that the research was conducted in the absence of any commercial or financial relationships that could be construed as a potential conflict of interest.

## Publisher's Note

All claims expressed in this article are solely those of the authors and do not necessarily represent those of their affiliated organizations, or those of the publisher, the editors and the reviewers. Any product that may be evaluated in this article, or claim that may be made by its manufacturer, is not guaranteed or endorsed by the publisher.
